# Electrochemical DNA Biosensors Based on Labeling with Nanoparticles

**DOI:** 10.3390/nano9101361

**Published:** 2019-09-23

**Authors:** Christos Kokkinos

**Affiliations:** Laboratory of Analytical Chemistry, Department of Chemistry, National and Kapodistrian University of Athens, 15771 Athens, Greece; christok@chem.uoa.gr; Tel.: +30-210-727-4312

**Keywords:** DNA, hybridization, biosensor, gold nanoparticles, silver nanoparticles, quantum dots, label, biosensor, stripping voltammetry, paper-based devices

## Abstract

This work reviews the field of DNA biosensors based on electrochemical determination of nanoparticle labels. These labeling platforms contain the attachment of metal nanoparticles (NPs) or quantum dots (QDs) on the target DNA or on a biorecognition reporting probe. Following the development of DNA bioassay, the nanotags are oxidized to ions, which are determined by voltammetric methods, such as pulse voltammetry (PV) and stripping voltammetry (SV). The synergistic effects of NPs amplification (as each nanoprobe releases a large number of detectable ions) and the inherent sensitivity of voltammetric techniques (e.g., thanks to the preconcentration step of SV) leads to the construction of ultrasensitive, low cost, miniaturized, and integrated biodevices. This review focuses on accomplishments in DNA sensing using voltammetric determination of nanotags (such as gold and silver NPs, and Cd- and Pb-based QDs), includes published works on integrated three electrode biodevices and paper-based biosystems, and discusses strategies for multiplex DNA assays and signal enhancement procedures. Besides, this review mentions the electroactive NP synthesis procedures and their conjugation protocols with biomolecules that enable their function as labels in DNA electrochemical biosensors.

## 1. Introduction

Deoxyribonucleic acid (DNA) is the carrier of genetic information and the basic material of biological heredity. Specific DNA sequences obtain a significant position in medical, food, and environmental analysis, as their detection provides practical ways to identify and diagnose a wide variety of infectious and inherited diseases [1,2,3,4,5]. Thus, innovative and sensitive procedures that engage different recognition and transduction platforms are requested for DNA detection. Via researchers’ efforts, numerous DNA-testing systems have been introduced, such as surface plasmon resonance, fluorescent and others assays based on the coupling of electrophoretic separations, and radioisotopic detection [5,6,7,8]. While these methodologies are considered the gold standards for DNA diagnostics, they are not in the position to cover the increase of requirements for point-of-care (POC) diagnostics, mainly due to the high cost and large size of their instrumentation. These limitations have forced the scientific community to search for alternative DNA sensors, which could offer simple operation as well as rapid, specific, sensitive, and multiplexed analysis using low-cost and portable equipment. An important ally in this effort is the use of electrochemical sensors, as they provide the above-mentioned features and, as a result, a plethora of smart bioanalytical devices suitable for POC analysis have been constructed over the past years [9,10,11]. Especially, the coupling of electrochemical sensors with micro- and nanoscale materials (i.e., carbon nanomaterials, magnetic microbeads, noble metal nanoparticles (NPs), and quantum dots (QDs)) has brought additional sensitivity and selectivity to DNA sensing [1,2,3,4,12,13,14,15,16].

Bioassays include the interaction between the analyte and a proper biological recognition compound, which interaction generates a measurable signal monitoring by a suitable transducer. Electrochemical biodevices are these sensors that integrate both the biological recognition and the electrode transducer into a single system. The DNA hybridization event is the foundation of all types of DNA detection platforms. According to DNA hybridization, a single-stranded oligonucleotide complementary to the target DNA is immobilized on a sensing area. Next, the sample which contains the target DNA is added and the DNA hybridization event is detected via changes in electrochemical parameters or the redox activity of electroactive labels [1,2,3,4,5,12,13,14,15,16,17]. While there are several label-free bioassays [18,19,20], the application of an appropriate label ensures higher selectivity and sensitivity, albeit at the expense of assay workflow simplicity.

Labels are molecules, ions, or atoms which serve as “barcodes” and purposefully interact with the target molecule or the biological recognition compound. The use of enzymes as labels is widespread [21,22,23], but lately many attempts have to do with their replacement with biohybrid nanoparticles, which afford unique advantages. Nanoscale materials present high stability, excellent conductivity, and capability in facilitating the electron transfer between the biomolecules immobilized on these materials and the electrode surface [3,4,12,13,14,15,16,17,24]. There are two main categories of NPs applied as electrochemical labels in DNA biosensing, and these are noble metal NPs and QDs. Gold and silver NPs (AuNPs and AgNPs) are the most widely used noble metal NPs, thanks to their stability, simple synthesis, and their ability scope for conjugation with biomolecules. QDs are NPs with size between 1 and 20 nm and composed of metal salts (such as PbX, CdX, ZnX (X:S, Se, Te)) and present exceptional electronic properties, as well as offer multiplex detection based on different redox potential associated to each metal ion [3,4,12,13,14,15,16,17,24].

In DNA electrochemical label-based bioassays, the target DNA interacts both with the capture oligonucleotide and with the recognition probe, which is conjugated with an appropriate NP label. Then, metal NP or QD labels are dissolved in an acidic media (e.g., HNO_3_, HCl) and the released cations are determined by a voltammetric technique, such as stripping voltammetry (SV). More specifically, in the case of SV, the released cations from metal NP or QD labels are preconcentrated on a suitable working electrode by reduction as the respective metal, and then, are determined after oxidation in the course of focuses on voltammetric scan. The voltammetric peak height is related to the concentration of the target DNA [15,16,17]. The synergetic effect of voltammetric preconcentration and the plethora of metal cations released from NP tags paves the way to ultrasensitive bioassays with sub-picomolar limits of detection [15,16,17]. In the case of AuNPs and AgNPs, differential pulse voltammetry (DPV) has also be applied as another simple detection mode [24].

This review presents a survey of electrochemical DNA biosensors using functional nanomaterials as labels, focusing on developments in integrated voltammetric transducers and paper-based sensors, as well as on architectures for signal amplification and multiplexed detection of DNA. The synthesis procedures of NPs and QDs and their conjugation with biomolecules is also discussed.

## 2. Synthesis and Modification of NPs for Labeling DNA Applications

**Figure 1:**
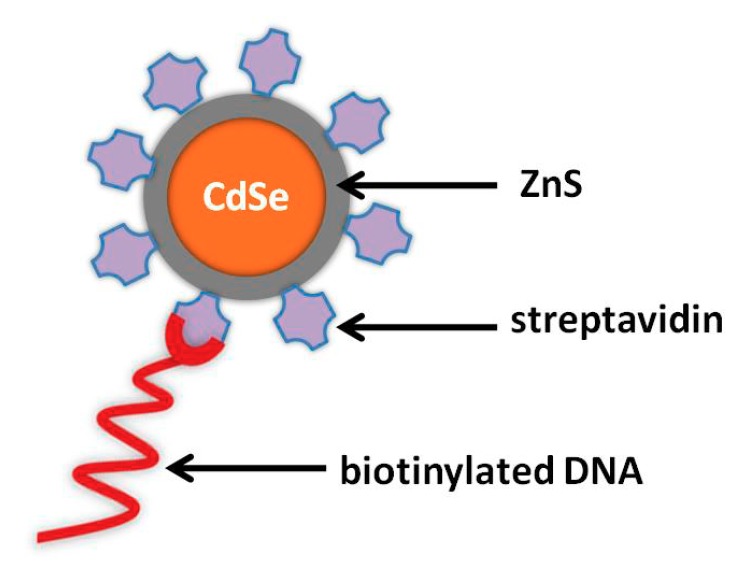
Schematic illustration of CdSe/ZnS QD modified with streptavidin and its conjugation with biotinylated oligonucleotide.

Nanoparticles can be synthesized following different synthesis procedures [4,13], the most important from an electrochemical point of view being gold and silver NPs and QDs (such as CdS, PbS, and ZnS). Their size distribution should be narrow in order to ensure high reproducibility during the electrochemical assays when applied as labels. Besides, NPs should be dispersed in an appropriate solvent, which prevents their agglomeration. Another important factor for metal NPs and QDs is their surface modification with proper groups (such as amino and carboxyl groups), allowing their conjugation with specific biomolecules (Figure 1). In this section, the most applied strategies of NP and QD synthesis and modification are described.

### 2.1. Synthesis of AuNPs and AgNPs

Numerous processes on the synthesis of NPs and QDs have been introduced and reviewed recently [4,13]. Regarding DNA applications, the typical synthesis of AuNPs relies on the reduction of Au(III) (from hydrogen tetrachloroacurate, HAuCl_4_) to Au(0) in the presence of a capping agent, such as sodium borohydride, D-glucose, or sodium citrate—which is the most applied [4,13,25,26,27,28]. According to these protocols, an aqueous solution of HAuCl_4_ is boiled in a conical flask under stirring. While the gold solution is refluxing, an aqueous sodium citrate solution or another capping agent is introduced in the flask and the solution color turns red, confirming the synthesis of the AuNPs. The suspension is allowed to cool, and synthesized AuNPs are separated via centrifugation [25,26,27,28].

The AgNPs are also synthesized by the chemical reduction of Ag(I) (e.g., AgNO_3_) in the presence of a capping agent such as NaBH_4_, resulting in a dark brown solution [29,30,31,32].

### 2.2. Synthesis of QDs

In QD-based DNA assays, the synthesis of QDs is based on different methodologies, depending on the desirable QD core [4,13,33,34,35,36,37,38,39,40]. In the case of CdTe synthesis, cadmium chloride, mercaptopropionic acid, and distilled water are mixed into flask. NaOH is added in order to adjust pH to 9.0, and then sodium tellurite and sodium borohydride are mixed with the above solution, and the resulting solution is deaerated with nitrogen and heated. Then, CdTe QDs are precipitated with ethanol and centrifuged [33,34]. In the case of CdSe QDs, cadmium acetate, oleic acid, and octadecne are placed in flask, heated, and degassed with N_2_. Next, selinium solution in trioctylphosphine is injected into the flask and the heating is continued. After cooling, QDs are formed and purified with chloroform and acetone, while they are dispersed in toluene. The oleic acid attached to the surface of QDs can be replaced with mercaptopropionic acid via heating, and the water-soluble QDs can be extracted by centrifugation [35,36]. The typical synthesis of CdS, PbS, and ZnS is based on dissolution of salts of cadmium, lead, or zinc and Na_2_S in separate sodium bis(2-ethylhexyl)sulfosuccinate/n-heptane mixtures. The two mixtures of heavy metal salts and Na_2_S are mixed and stirred under inert conditions to yield the CdS, PbS, or ZnS nanoparticles. The QDs are capped by adding cysteamine and sodium 2-mercaptoethane sulfonate. The resulting QDs are obtained by evaporating the heptane and washing with pyridine, hexane, and methanol [37,38,39,40].

### 2.3. Functionalization of Metal NPs and QDs with Biomolecules

The binding of oligonucleotides onto the surface of NPs can be conducted via biotin–(strepta)avidin linkage. For instance, QDs are functionalized with carboxyl groups by suspending QDs in mercaptoundecanoic acid. The excess thiol is removed with centrifugation, followed by dispersion in phosphate buffer solution (PBS) containing N-(3- dimethylamminopropyl)-N′-ethylcarbodiimide hydrochloride(EDS) and N-hydroxysulfosuccinimid sodium salt (NHS). After stirring, the mixture is centrifuged and then dispersed in PBS. After that, the desired protein (steptravidin, avidin, or biotin) is added and mixed. The QD–protein conjugates are collected by centrifugation and resuspended in PBS or Tris-HCl [37,40,41,42,43,44].

Other methods to attach oligonucleotides onto NPs are: (i) Through terminal thiol groups by mixing NPs with thiolated oligonucleotides, and (ii) conjugation of aminated DNA with carboxylated NPs. For example, PbS, CdS, or ZnS QDs are mixed with thiolated oligonucleotides under stirred and inert conditions. To this mixture, the suitable salts are added to generate NaCl and PBS. Then, the mixture is dialyzed against NaCl and PBS containing sodium azide [38,45]. In the case of conjugation of aminated DNA with carboxylated NPs, for instance, carboxylated CdS QDs are dissolved in water, the pH is adjusted to 7.3, and NHS and EDC are dissolved in the suspension under stirring. After that, aminated DNA is added dropwise to the mixture and incubated at 4 °C. The QD-labeled DNA is obtained after centrifugation and dispersion into appropriate buffer [39].

## 3. Electrochemical Determination of NPs and QDs Labels

Nanoparticles are applied in many bioanalytical formats serving as electrode modifiers, acting either as electron wires or as electrochemical catalysts, as carriers of labels, as seeds for metal deposition, and as electrochemical labels. The present review focuses on the application of NPs as labels in the construction of ultrasensitive DNA electrochemical biosensors, while the other functions of NPs have been excellently reviewed recently [3,4,12,24,46]. When NPs are applied as quantification labels, the electrochemical signal which emanates from the NPs is measured by voltammetry (SV or pulse voltammetry (PV)). Both voltammetric techniques incrementally change the applied working electrode potential in a step manner. After the interaction between the target DNA and NP-conjugated probe, the QD or metal NP labels are transformed to their respective ions through their acidic dissolution, and they are determined either by direct voltammetry or by SV. In the case of SV, the released cations by NP labels are preconcentrated on the working electrode by reduction, and then they are detected after their oxidation [17,46]. DNA biosensors using voltammetric detection modes take advantage of the duplex signal amplification provided by the association of voltammetric preconcentration step and the plethora of individual metallic ions liberated from metal NP and QD tags.

### 3.1. Voltammetric Determination of AuNPs Labels

**Figure 2:**
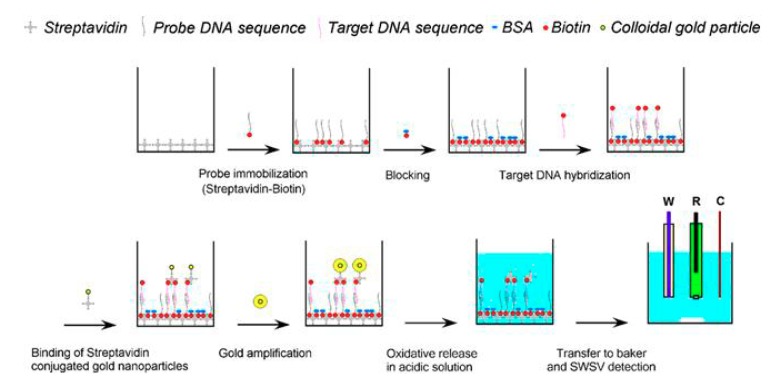
Schematic illustration of DNA bioassay using AuNPs as labels and their SWSV determination at glassy carbon working electrode. Biotinylated DNA probe immobilized on a streptavidin-coated microwell, which was hybridized with biotinylated target while streptavidin-conjugated AuNPs bound on target DNA, followed by dissolution in acid solution and detection by SWSV method. Reproduced from [48], with permission from Elsevier, 2019.

The determination of Au ions is carried out only at carbon-based transducers, as the potential of gold oxidation is more positive than the oxidation potentials of mercury, bismuth, and tin, which are the commonest electrode materials in SV and QD assays. The electrochemical detection of AuNP tags can be performed through their oxidative dissolution in a HBr/Br_2_ solution by applying potential of approximately +1.2 V (vs. Ag/AgCl). The obtained Au(III) cations are preconcentrated by reduction onto the electrode surface and subsequently determined by SV [28,47,48,49]. For example, a biotinylated oligonucleotide probe DNA was immobilized in a streptavidin-modified microwell and the free were blocked with bovine serum albumin. The biotinylated target DNA was then added and hybridized with the capture probe DNA and streptavidin-modified AuNPs used as labels. The AuNPs labels were dissolved in HBr/Br_2_ solution and DNA determination was performed using square wave SV (SWSV) mode and glassy carbon working electrode, while the limit of detection (LOD) was 130 aM (Figure 2) [48].

Another procedure for the determination of AuNPs is their electrochemical oxidation in HCl. This protocol includes a preoxidization step in order to oxidize AuNPs to AuCl_4_, and the produced AuCl_4_ are determined by voltammetry [27].

#### Signal Enhancement Strategies Using AuNPs Labels

In order to enhance the sensitivity of AuNP-based DNA bioassays, different methodologies have been adopted, such as the conjugation of AuNPs with latex microspheres [28,49] and/or with magnetic beads (MBs) [27,49,50]. As ‘tracer amplification’, Ag deposition on the AuNPs after hybridization is also applied, and an enhanced electrochemical signal attributable to Ag is obtained; SV determination is used for this AuNPs/Ag enhancement platform [51,52].

**Figure 3:**
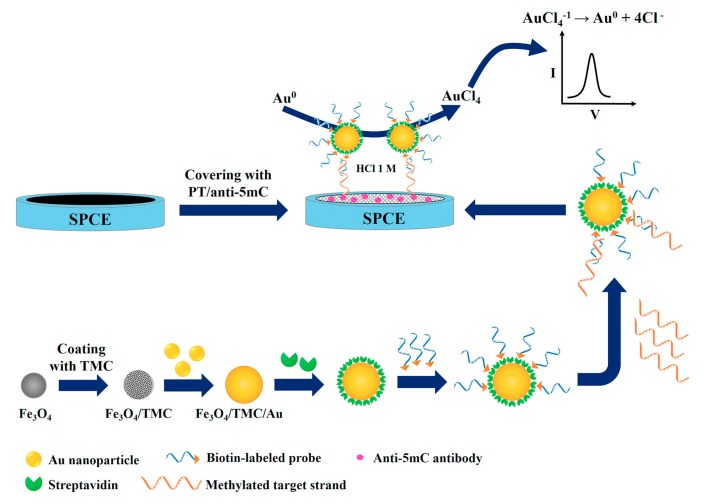
Schematic illustration of DNA bioassay using MBs/N-trimethylchitosan/AuNPs as tags to label DNA probe. DPV was used for the determination of DNA of RASSF1A tumor, applying electrochemical oxidation in HCl and screen printed carbon working electrode. Reproduced from [27], with permission from Elsevier, 2019.

MBs are particles which are composed of a paramagnetic or superparamagnetic core (mainly based on different iron oxide forms), and their surface can be functionalized with capture biomolecules. The application of MBs in DNA assays offers noteworthy advantages, as the target analyte is preconcentrated on the surface of the modified MBs, while the application of magnetic field separates the MB–analyte complex from the matrix of the sample, leading to the minimization of matrix effects and the amplification of the bioassay selectivity. For example, a novel chip for the detection of RASSF1A tumor suppressor gene methylation has been developed using Fe_3_O_4_/N-trimethylchitosan/AuNPs as tags to label DNA probe. DPV was employed for the quantitative analysis of DNA with a LOD of 2 fM, by applying HCl on the surface of modified screen printed carbon electrode. The electrochemical detection involved oxidation of the AuNPs at +1.25 V (vs.Ag/AgCl) and reduction to Au(0) (Figure 3) [27].

Another ultrasensitive electrochemical DNA bioassay has been developed for *Vibrio cholerae* DNA, applying MBs as the biorecognition substrate and AuNP-loaded latex microspheres as labels [49]. The biorecognition surface was prepared by immobilizing specific biotinylated capturing DNA probes onto streptavidin-conjugated MBs. The fabrication of labels involved loading of AuNPs onto polyelectrolyte film-coated poly(styrene-co-acrylic acid) latex microspheres. The target DNA was sandwich-hybridized to MB- -captured probes and labeled with latex–AuNPs. The latex–AuNP-tagged hybrid-bound MB complexes were resuspended in HBr/Br_2_ to chemically dissolve the AuNP tags as Au(III), which were determined by DPSV at screen printed carbon working electrode.

### 3.2. Voltammetric Determination of AgNPs Labels

AgNPs have also been used as labels in DNA biosensors [29,30,31]. AgNP tags are easily produced by in situ metallization of silver. An example of this protocol is the development of a voltammetric DNA biosensor for the detection of sequence-specific DNA [30]. Initially, thiolated peptide nucleic acid probes were immobilized onto gold surface and the target DNA was hybridized. Next, hematin were added to the hybridized heteroduplexes. The attached hematin molecules acted as a catalyst, boosting the reduction of Ag ions in the presence of catechol, leading to the in situ deposition of AgNPs onto the electrode. The deposited silver nanoparticles were electrochemically stripped into KCl solution and measured by SWV with an LOD of 62.41 aM.

**Figure 4:**
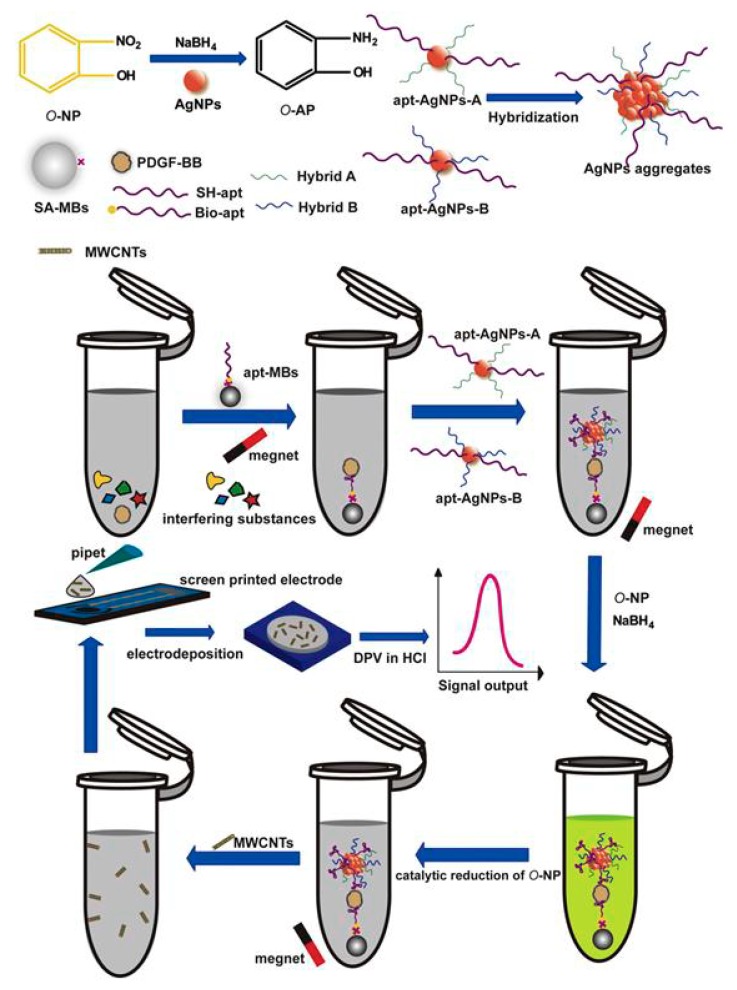
Schematic representation of DNA biosensor using AgNP labeling, multiwalled carbon nanotube modifiers of screen-printed electrode and MBs for enhanced sensitivity and selectivity. Reproduced from [31], with permission from Elsevier, 2019.

In addition, AgNP labeling can be combined with MBs for enhanced sensitivity and selectivity. An interesting biosensor for the determination of platelet-derived growth factor BB PDGF-BB was based on magnetic separation, AgNP labeling, and multiwalled carbon nanotube (MWCNT) modifiers of screen-printed electrode surface [31]. The capture probe was MBs functionalized with aptamers (apt–MBs), and AgNPs modified with aptamers were applied as labels. In the presence of PDGF-BB, the apt–MBs and the AgNPs formed a sandwich-like complex, followed by the adding of NaBH_4_ and o-nitrophenol. Thanks to the catalysis of AgNP aggregates conjugated in the complex, the o-nitrophenol was reduced by NaBH_4_ to o-aminophenol, which was electro-co-deposition with MWCNTs on the electrode, forming a conducting nanocomposite. The signal in the PDGF-BB biosensor was obtained via measurement of the poly(o-AP-MWCNTs) film on the screen printed electrode (Figure 4) [31].

### 3.3. Voltammetric Determination of QDs Labels

As QDs are composed of a metallic core of Pb, Cd, Zn, their detection can be carried out through their acidic dissolution (e.g., in HNO_3_, HCl), and the liberated cations determined by SV. For the SV determination of these cations, bare carbon electrodes can be applied as transducers [34,53] but for higher sensitivity, the determination of Pb(II), Cd(II), and Zn(II) is preferably conducted at mercury-, bismuth-, antimony-, and tin film electrodes [33,35,36,37,38,39,40,41,42,43,44]. The most typical process for the production of metal film sensors is the in situ electroplating on carbon or gold surfaces [33,35,36,38,39,40,41,42,43,44]. According to in situ electroplating, cations of mercury, bismuth, antimony, or tin are introduced into the analysis solution, and the metallic film is formed on the sensor surface during the preconcentration step of the target ions. Besides, the sputtering deposition process can be applied for the construction of film electrodes, as well as loading of the sensor with a Bi-precursor compound [37,54,55].

#### 3.3.1. Mercury-Based Sensors for QD-Based DNA Assay

Mercury electrodes have been extensively applied for voltammetric determination of trace metals and QD labels in DNA bioassay. In terms of electroanalytical performance, Hg sensors are eminent for metal analysis at trace level, thanks to their sensitivity, reproducibility, and wide cathodic polarization. Numerous QD-based DNA bioassays have been introduced using mercury film electrode as transducer [33,35,36,38,39,40,41,42,43], and the mercury film is created via in situ electroplating protocol on carbon or gold electrodes. Recently, Sun et al. developed a DNA assay in microwells, which was based on a target-induced strand displacement reaction with blocker DNA (labeled with CdS QDs) from a biotinylated hairpin DNA [39]. According to this assay, a hairpin-blocker DNA was immobilized on the surface of the microwell through biotin–streptavidin interaction. On addition of target DNA, the CdS-labeled blocker DNA was displaced by target DNA from the hairpin-blocker to form a new target-blocker DNA. Then, Cd(II) was released from the QDs using HNO_3_, and determined by SV applying an in-situ formed Hg film sensor, and the LOD was 1.2 pM.

An interesting QD-based electrochemical biosensor was developed in 2018 for human determination of telomerase activity at the single-cell level by Li et al. [42]. For this purpose, a thiol-modified capture DNA was attached on Au surface via the Au–sulfur bond. The presence of telomerase enabled the addition of the telomere repeats to the 3’ end of the primer, accompanied by the incorporation of abundant biotins in the extension product. The hybridization of extension product with the capture oligonucleotide and the reaction with streptavidin-modified quantum dots caused the concentration of a large number of quantum dots onto the sensor through streptavidin–biotin interaction. The liberated Cd(II) from acidically-dissolved quantum dots was determined by SV at Hg film electrode in situ formed on a glassy carbon surface.

#### 3.3.2. Bismuth- and Tin-Based Sensors for QD-Based DNA Assay

Mercury, despite its unique electroanalytical properties, is toxic and bioaccumulates in tissues. The hazards, which arise from the disposal and handling of Hg, have forced the reduction of Hg use in laboratories. In the quest for eco-friendly electrode materials, the research efforts have been focused on developing alternative “green” voltammetric sensors. Bismuth, antimony, and tin provide excellent electrochemical characteristics and can serve as mercury-free sensors [56,57,58,59]. Regarding QDs-based voltammetric DNA bioassays, bismuth and tin transducers haven been applied. Except for in situ electroplating protocols for the production of bismuth film sensors [41,60,61,62], the loading of the transducer with a Bi-precursor compound (such as bismuth citrate) and the sputtering fabrication processes have been introduced by our group [37,54]. In the case of tin electrodes, only microfabricated sensors have been used as transducers in QD-based DNA bioassays [55,63]. For instance, the detection of DNA sequence of the C634R mutation has been carried out at a graphite screen-printed electrode modified with bismuth citrate [37]. The Bi-citrate acted as a precursor compound for the in situ formation of Bi film on the surface of the working electrode. The precursor compound was reduced to metallic Bi at the same time with the deposition of the cation released from QD labels on the sensor surface. For the DNA determination, biotinylated DNA probes reacted with streptavidin-modified PbS QDs. The SV detection of released Pb(II) was carried out at the graphite electrode, modified with the bismuth precursor compound, and the LOD was 0.03 pM of DNA.

**Figure 5:**
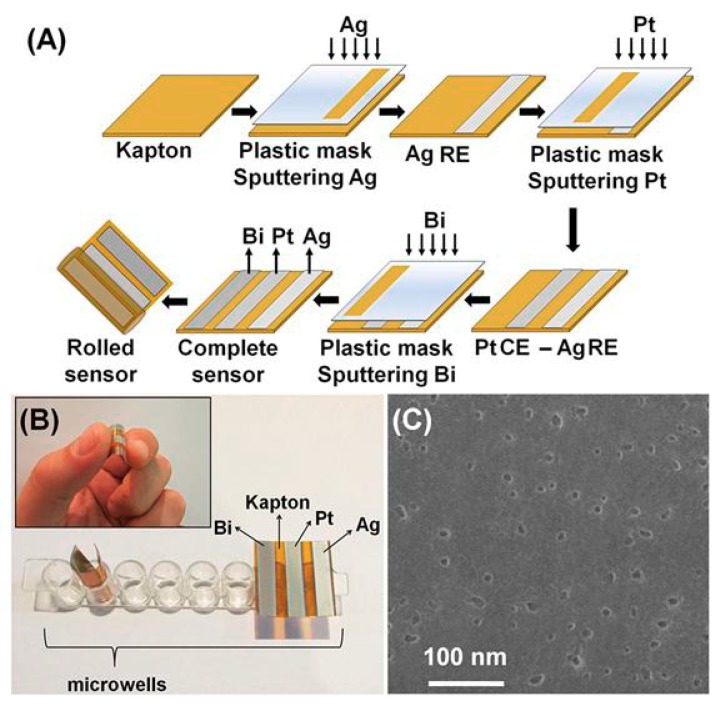
(**A**) The fabrication steps of a flexible bismuth sensor on polyimide substrate through sputtering deposition process. (**B**) Photographs of the sensors. (**C**) A field emission scanning electron microscope image of streptavidin-modified PbS QDs. Reproduced from [54], with permission from American Chemical Society, 2019.

A flexible Bi-based sensor suitable for QD-based voltammetric bioassays directly in microtitration wells has been also fabricated [54]. The microdevice was composed of bismuth, silver, and platinum thin films which were deposited by sputtering on a thin polyimide film (Figure 5). The DNA assay was developed in microtitration wells, where the complementary DNA probe hybridized with the biotinylated target oligonucleotide, while streptavidin-modified PbS QDs were used as tags. The flexible sensor was rolled and inserted into the microtitration wells to determine the acidically-released Pb(II) from QDs in situ by SV. Thanks to the in situ voltammetric determination directly in the microtitration wells, the sample dilution was minimized, leading to the reduction of the LOD at femtomolar levels.

Recently, tin film electrodes produced on silicon wafer by microengineering processes (sputtering and photolithography) were used as transducers for voltammetric determination of Cd(II) liberated from QD tags, enabling the detection of DNA at nanomolar levels [55]. For the DNA assay in microwell, a capture complementary DNA was hybridized with the biotinylated target DNA, followed by labeling with streptavidin-modified Cd-based QDs. Comparative studies among in situ electroplated bismuth- and mercury film electrodes and a microfabricated tin sensor show that the tin microsensor presented about 3-fold higher sensitivity to the stripping voltammetric determination of cadmium cations.

#### 3.3.3. Signal Enhancement Strategies Using QD Labels

**Figure 6:**
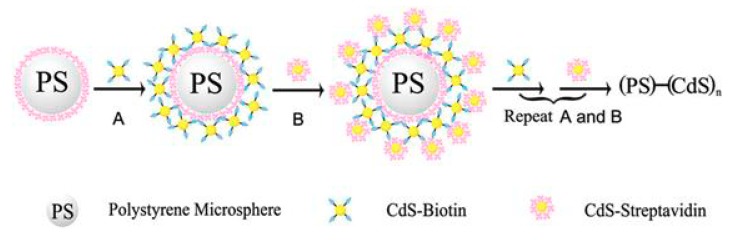
Schematic illustration of the formation of the PS–(CdS)n assemblies. Reproduced from [65], with permission from American Chemical Society, 2019.

The sensitivity of QD-based voltammetric DNA assays can be further enhanced by the application of MBs [38,53,60,62,64,65], QD layer-by-layer assembled polystyrene microsphere (PS) composite [61,64,65], and carbon nanotubes [35,40] (Table 1). Carbon nanotubes assist electrochemical redox reactions by virtue of their high conductivity [66]. According to the approaches of the assembled labels, the surface of each microbead is loaded with a large number of QDs and thus, the quantity of QDs in every binding event is amplified, and the signal is increased. An interesting example of assembled labels has been described by Xiang, combining MBs as a biorecognition platform and QDs–PS beads as labels [65]. The target DNA was sandwich-hybridized with the immobilized capture probes on the surface of MBs and, with the signaling probes, conjugated to the QDs–PS beads. The QDs–PS beads were produced by the interaction of streptavidin and biotin-modified CdS QDs, respectively, onto the surfaces of PS microsized particles (Figure 6). Thanks to the signal enhancement by the plethora of QDs involved in every DNA binding event, the LOD was 0.22 fM.

**Figure 7:**
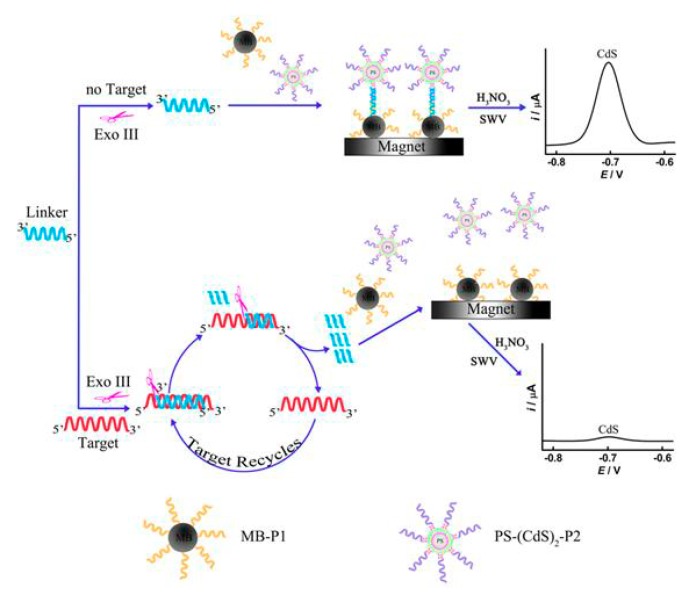
Schematic image of the duplex amplification process using enzymatic recycling and the layer-by-layer PS–(CdS)_2_ assemblies. Reproduced from [64], with permission from Elsevier, 2019.

Another example of signal enhancement is the combination of the enzymatic target recycling method with the QD layer-by-layer assembled labels onto PS beads [64]. The enzyme-based catalytic DNA recycling procedure resulted in the use of each target DNA sequence multiple times, and thus, the analytical signal was direct amplified. The combination of these two successful signal amplification procedures offered a LOD of 5 fM of the target DNA sequences. In the absence of the target DNA, the enzyme was inert to the linker strands. After thermal deactivation of the enzyme, the linker strands hybridized with the complementary DNA on the PS–(CdS)_2_ assemblies and the MBs. After magnetic isolation, the PS–(CdS)_2_ assemblies coupled to the MBs were dissolved in HNO_3_ and the liberated Cd(II) were determined by SWASV. On the contrary, the existence of the target DNA hybridized with the linker strands to generate DNA duplexes and the enzyme exclusively digested the linker strands to liberate the target DNA. The liberated target DNA again hybridized with other undigested linker strands and triggered another target recycling cycle with the aid of the enzyme (Figure 7). These catalytic reactions offered the digestion of more and more linker strands, which in turn caused the capture of less PS–(CdS)_2_ assemblies by the MBs and resulted in an amplified suppression of the Cd(II) response.

## 4. Multiplexed Detection of DNA Sequences Exploiting NPs as Labels

QDs labeling offers the potential to develop multiplexed determination architectures, based on the separated oxidation potentials of the different QD tags [17,38,67,68,69,70]. On the other hand, metal NP-labeled voltammetric transducers are not able to perform multi-analyte electrochemical bioassays in a single run applying different NP labels. This is attributed to the fact that the oxidation potential values of frequently applied NPs (Au, Ag) are relatively close to each other and the respective current peaks can potentially overlap. Therefore, there are two key formats for multiplexed determination of biomolecules. The first uses different types of specific quantum dots (e.g., PbS, CdS, and ZnS) to label two or more different biomolecules. In this case, the determination is conducted simultaneously at a single working electrode, and is based on the different oxidation potentials of Pb(II), Cd(II), and Zn(II) liberated after the of quantum dots. This approach for DNA assay was suggested by J. Wang’s group [38]. A second multiplexing strategy, which is fit to metal NP-labeled electrochemical biosensors, is based on the use of single type of metal NPs and array of electrodes utilizing spatially-separated working electrodes. In this process, the determination is carried out at each spatially-separated electrode, in either a parallel or serial mode. Application of the second multiplexing strategy is limited to protein analysis [71,72], and thus, in this section, only the multiplexed detection of DNA using QD-labeling is described (Table 1).

**Figure 8:**
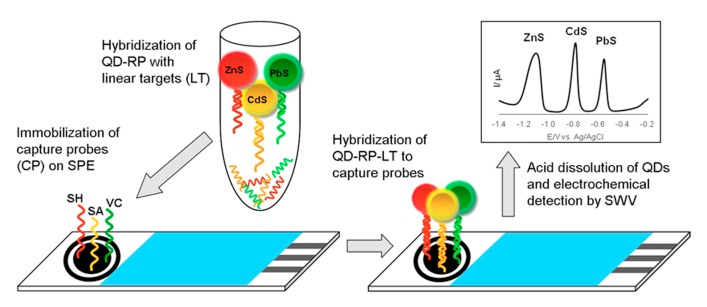
Schematic illustration of sandwich DNA hybridization assay for simultaneous determination of the three pathogens using PbS, CdS, and ZnS QDs. Reproduced from [67], with permission from Elsevier, 2019.

A smart biosensor for the simultaneous detection of sequences of *Vibrio cholerae*, *Salmonella* sp., and *Shigella* sp., developed by Vijian et al. [67]. CdS, ZnS, and PbS QDs conjugated with DNA probes that were specific to each target analyte were used as labels and sandwich-hybridization assays were applied (Figure 8). Electrochemical determination of Cd(II), Zn(II), and Pb(II) liberated from QDs was performed using SWSV at a screen-printed carbon working electrode with an in situ electroplated formed Bi film offered LODs at attomolar scale.

Signal amplification methodologies can be also coupled with multiplexed bioassays [38,67,70]. For example, a voltammetric biodevice for the simultaneous determination of the protective antigen A gene of *Bacillus anthracis* and the insertion element gene of *Salmonella* enteritidis was published [70]. The bioassay was based on three nanoparticles: AuNPs, MBs, and QDs (CdS and PbS). The AuNPs were attached to the first target-specific DNA probe, which recognized one end of the target DNA sequence, and QDs functionalized with DNA, which acted as labels. The MBs were coated with the second target-specific DNA that can recognize the other end of the target gene. After interaction of NPs with the target DNA, the use of magnetic field separated the sandwich structure from the unreacted compounds. Next, the nanoparticle tracers were dissolved in acid, and the Cd(II) and Pb(II) ions were determined by SV at a Bi film carbon screen-printed electrode.

**Figure 9:**
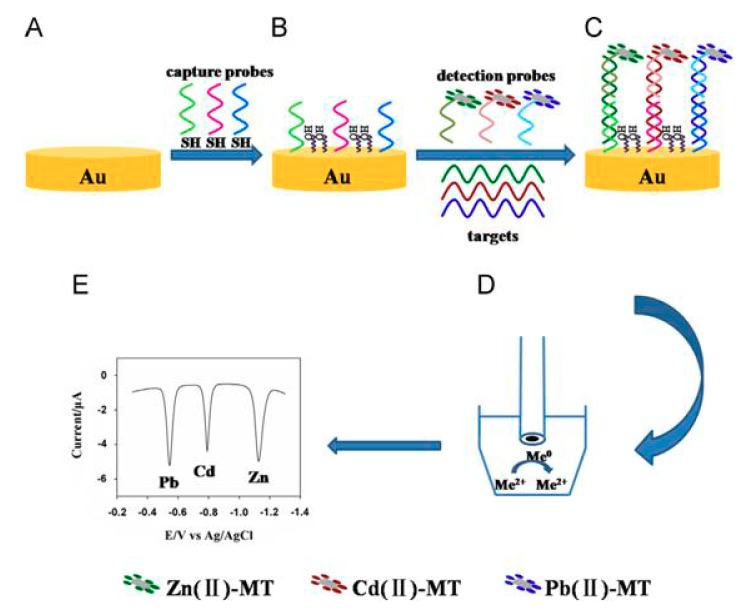
Schematic illustration of simultaneous determination of three DNA targets using encoding metal ions. (**A**) Au substrate. (**B**) Immobilization of the capture probes. (**C**) Hybridization events. (**D**) Determination of released metal ions. (**E**) SWSVs of Pb(II), Cd(II), and Zn(II). Reproduced from [69], with permission from Elsevier, 2019.

Another interesting multiplexing bioassay for the simultaneous voltammetric determination of multiple DNA targets was based on the application of different encoding metal ions as labels [69]. DNA/metallothionein conjugates which were attached with different metal ions were used as detection probes. The DNA targets were hybridized with the probes and the three encoding metal ions (Zn(II), Cd(II) and Pb(II)) were detected with SV at an in situ electroplated bismuth film on glassy carbon electrode (Figure 9).

## 5. Paper-Based Devices for DNA Sensing Using NPs as Labels

Paper is an excellent material for the performance of bioassays, as it is widely available, very cheap, hydrophilic, safe, disposable, and biocompatible. The surface of paper can be easily functionalized or patterned and presents high adsorptive properties for biomolecules and nanomaterials. Besides, paper allows the transport of liquids via capillary action, thus acting as an autonomous microfluidic pumping system, without the necessity for external pumps. Nevertheless, despite its unique advantages in biosensing, the field of electrochemical paper-based analytical devices (ePADs) applying metal nanoparticles and quantum dots as tags and their votlammetric determination still remains unexplored, since only a few ePADs have been developed for DNA sensing [32,63,73].

**Figure 10:**
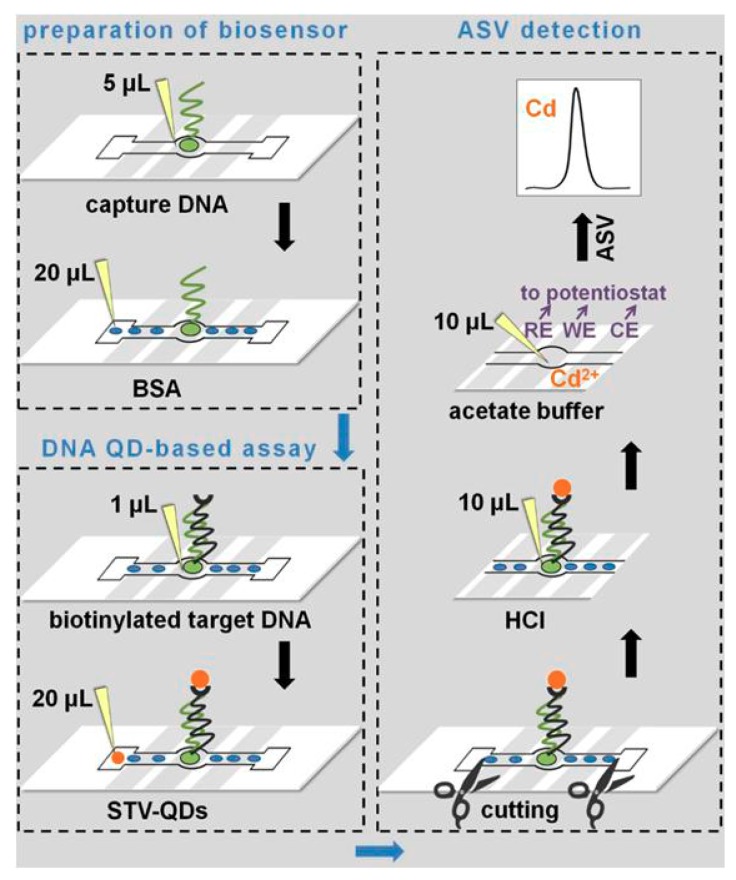
Schematic illustration of the development of paper-based DNA biosensor fabricated through wax-printing and sputtering. Reproduced from [63], with permission from American Chemical Society, 2019.

A microfabricated ePAD for the voltammetric determination of DNA (related to the Multiple Endocrine Neoplasia Type 2) using CdSe QDs as label has been introduced by Kokkinos et al. [63]. The ePAD was patterned by wax-printing and featured an assay zone connected to an inlet zone and a sink via grooved channels for increased flow. On the reverse side of the paper, an electrochemical cell was formed by the deposition of sputtered metal nanofilms (tin, platinum, and silver as the working, counter, and reference electrode, respectively). The DNA assay involved immobilization of capture complementary oligonucleotide, hybridization with biotinylated target DNA, and labeling with streptavidin-modified Cd-based quantum dots (Figure 10). The liberated cadmium cations from QDs was measured by SV at the tin film sputtered electrode. The target oligonucleotide was determined at levels as low as 0.11 pM requiring sample volumes as low as 1 μL and the cost of the ePAD was $0.11.

**Figure 11:**
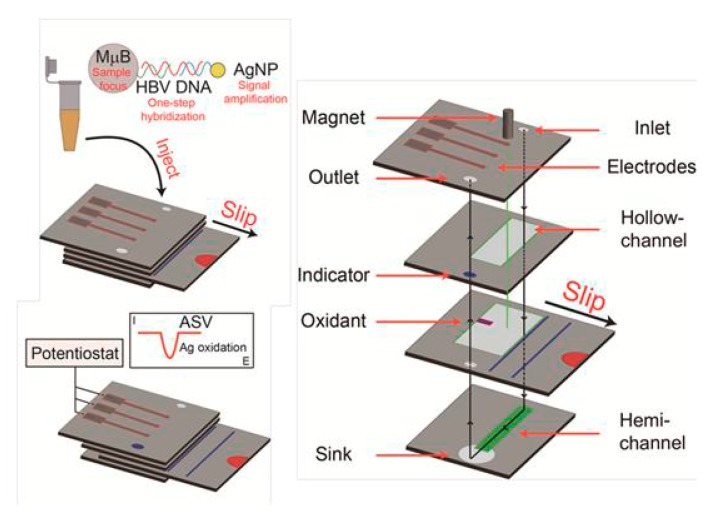
Schematic illustration of the development of paper-based biosensor the detection of DNA sequence from the hepatitis B virus, applying AgNP labeling and MBs as capture probes. Reproduced from [32], with permission from American Chemical Society, 2019.

Another interesting ePAD was fabricated by paper folding for the detection of DNA sequence from the hepatitis B virus (HBV) [32]. The design of ePAD combined paper folding assembly, the open structure of a hollow-channel to accommodate microparticles, and a convenient slip layer for timing incubation steps. Two steps of amplification were applied via AgNP labeling and MBs as capture probes (Figure 11). The cost of the device was $0.36.

**Figure 12:**
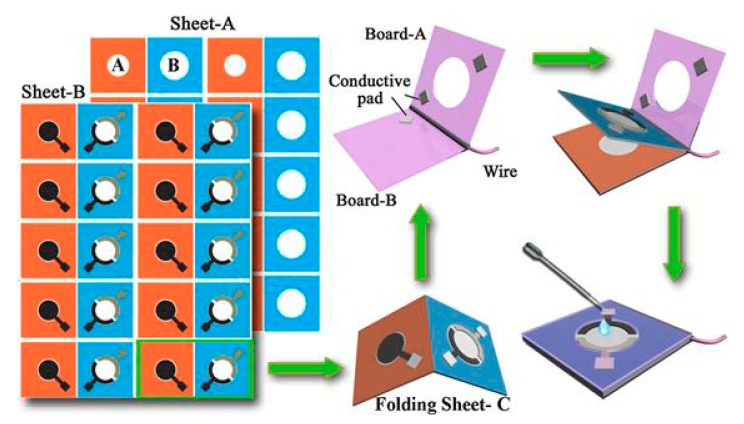
Schematic illustration of the fabrication and DNA assay procedure of the folding paper-based sensor device. Paper sheets were patterned in bulk using a wax printer and the three electrodes were screen-printed on wax-patterned sheets. Then, the sheet was cut to rectangular paper devices. Reproduced from [73], with permission from Elsevier, 2019.

Besides, a folding paper device for DNA sensing was introduced by Lu et al. [73]. The production procedure of the sensor consisted of wax-printing, baking the wax-patterned sheet, screen-printing electrodes, followed by cutting (Figure 12). The device was modified with AuNPs and graphene in order to achieve an efficient DNA immobilization, and the detection based on a sandwich assay applying Au nanoporous particles as labels. The LOD for target DNA was 0.2 fM.

## 6. Conclusions

The increasing requirements for very sensitive DNA assays have led to the production of electrochemical biosensors based on nanomaterials. In contrast to other methods, namely spectroscopy and chromatography, electrochemical techniques are significantly cheaper, simpler, and easier for miniaturization, establishing them suitable for POC analysis. On the other hand, nanomaterials present unique characteristics such as small dimensions, high surface-to-volume, and robustness, while they can functionalize with specific biomolecules such as antibodies, DNA, developing nanotags for various applications. Thus, the combination of electrochemistry and nanomaterials has paved the way to the production of highly selective and sensitive biosensors. This review explores the advantageous features of these architectures, highlighting the development of DNA electrochemical biosensors applying noble NPs and QDs as labels. There are two key features of nanolabel-based DNA biosensors: (i) The simultaneous multiplexing detection by combining different QD labels (such as CdS, PbS, and ZnS), and (ii) the multiple amplification strategies, utilizing two or more types of materials, such as magnetic beads and polystyrene microsphere.

Nevertheless, despite numerous successful proof-of-principle applications, some issues have not been yet addressed in the mission for practical biosensors and their commercialization. Besides, the integration of voltammetric DNA biosensors into paper-based microfluidic platforms with the inclusion of nanomaterials for determination requests to be expansively explored in future, as only a few ePADs have been reported in the relative literature. The production of these units would offer simpler, cheaper biosensors. Although NPs have been widely applied in electrochemical DNA biosensing, opportunities still exist. More specifically, novel materials which have been freshly introduced, such as graphene quantum dots and carbon dots [74,75], seem to be extra tools for developing more sensitive and selective electrochemical DNA sensing systems. Finally, two major challenges are the automation of DNA sensors and the data analysis by smartphones. Microfludic systems, in which all the assay steps are conducted on-line, look perfect for automation DNA purposes. In addition, the development of very small sized potentiostant which can be coupled with smartphones [76] would be very useful for the data manipulation of DNA microsystems and would offer the promise of simple and easy on-site applications requiring extremely small volumes.

## Figures and Tables

**Table 1:** Examples of signal amplification and multiplex strategies in DNA detection using QD labels and SV.

Electrode	Analyte	Signal Amplification	QDs	Reference
MFE plated in situ on GCE	35S promoter of cauliflower mosaic virus	CNTs	CdSe	[35]
MFE plated in situ on GCE	Single DNA target	CNTs/AuNPs/MBs	CdSe–CdS	[36]
MFE plated in situ on GCE	Multiple DNA targets	MBs	ZnS, PbS, CdS,	[38]
MFE plated in situ on GCE	Single DNA target	CNTs	CdS	[40]
Carbon SPCE	Cystic-fibrosis-related DNA sequence	MBs	CdS	[53]
BiFE plated in situ on GCE	Ochratoxin A and fumonisin B1 in maize	MBs	CdTe, PbS	[60]
MFE plated in situ on GCE	HPV-16	PS	CdTe	[61]
MFE plated in situ on GCE	Single DNA target	PS/MBs	CdS	[64]
MFE plated in situ on GCE	*Escherichia coli* uropathogens	PS/MBs	CdS	[65]
BiFE plated in situ on SPCE	Sequences of *Vibrio cholerae*, *Salmonella* sp, *Shigella* sp.,	-	PbS, CdS, ZnS	[67]
HMDE	Virus of H5N1 chains	MBs	PbS, CdS, ZnS	[68]
BiFE plated in situ on GCE	Multiple DNA targets	-	MT–Pb, MT–Cd, MT–Zn	[69]
BiFE plated in situ on SPCE	Gene of *Bacillus anthracis* and gene of *Salmonella* enteritidis	MBs	PbS, CdS	[70]

GCE: Glassy carbon electrode; SPCE: Screen-printed carbon electrode; QDs: Quantum dots; MT: ssDNA/metallothionein; AuNPs: Gold nanoparticles; CNTs: Carbon nanotubes; BiFE: Bismuth film electrode; MFE: Mercury film electrode; HMDE: Hanging mercury drop electrode; MB: Magnetic beads; PS: Polystyrene beads.
